# Updates to TRACK_TEST and TRACK_VISION Computer Programs

**DOI:** 10.3390/polym13040560

**Published:** 2021-02-13

**Authors:** Dragoslav Nikezic, Jelena M. Stajic, Kwan Ngok Yu

**Affiliations:** 1 Department of Mathematical Science, State University of Novi Pazar, 36300 Novi Pazar, Serbia; nikezic@kg.ac.rs; 2 Department of Science, Institute for Information Technologies Kragujevac, University of Kragujevac, 34000 Kragujevac, Serbia; stajicjelena11052012@gmail.com; 3 Department of Physics, City University of Hong Kong, Tat Chee Avenue, Kowloon Tong, Kowloon, Hong Kong, China

**Keywords:** solid-state nuclear track detectors, SSNTDs, track development, track profile, optical appearance

## Abstract

The computer programs TRACK_TEST and TRACK_VISION were previously developed to model profiles and optical appearances of tracks developed in solid-state nuclear track detectors. The programs were based on a track development model that involved the bulk etch rate *V_b_* and the track etch rate *V_t_* or the *V* function (i.e., *V_t_*/*V_b_*). The present work reported our work to update and modify these two programs. In the revised TRACK_TEST, two new *V* functions were added and enabled. Sample results for the CR-39 detector obtained using the three original and the two new *V* functions were compared. Discrepancies were within ~10% and <14% for incident alpha-particle energies of 1 MeV and >1 MeV, respectively. Another major revision of TRACK_TEST was to enable calculations for the Makrofol detector. In the revised TRACK_VISION, the two new *V* functions, as well as the option for the Makrofol detector, were also added. The experimental results on the Makrofol detectors were obtained (irradiated with 3.6-MeV alpha particles under normal incidence and then etched to achieve a removed detector thickness of 30 μm) for comparisons with the modeled results using the revised TRACK_VISION. The track diameters obtained from the experiment and model were 24.7 and 23.2 μm, respectively. Moreover, a bright area in the central parts, together with an outer dark ring, were present in both the simulated and experimental tracks. The track-opening diameters and the general optical appearances of the tracks were in good agreement.

## 1. Introduction

Solid-state nuclear track detectors (SSNTDs) have been widely used for radon measurements through counting and/or characterizing the tracks created by the incident alpha particles emitted form radon gas and its progeny and by the subsequent chemical etching (see, e.g., References [1,2,3,4,5,6,7]). When a heavy, charged particle passes through an SSNTD, a cylinder consisting of free chemical radicals and other chemical species is formed along the trajectory, and this cylinder is referred to as a “latent track”. When the irradiated SSNTD is immersed into an aggressive solution called an etchant, the latent track will undergo more intensive chemical reactions compared to the unirradiated areas, leading to formation of a “track” that can be observed under an optical microscope.

Among the most widely used SSNTDs are polyallyldiglycol carbonate (PADC) films (commercially available as CR-39 detectors), cellulose nitrate films (commercially available as LR 115 detectors) and polycarbonate films (commercially available as Makrofol detectors). Different aspects of SSNTDs were reviewed by Nikezic and Yu [8]. Optical microscopes are commonly used for counting or characterizing alpha-particle tracks. It is also noted that optical features of the etched tracks, such as their optical appearances (bright and dark areas, etc.) can provide useful information on track parameters, such as track depths, measurements of which using common optical microscopes remain difficult.

To tackle these challenges, our group developed the computer program TRACK_TEST [9] in 2006 to theoretically model alpha-particle track profiles, which enabled the determination of parameters, including the lengths of the major and minor axes, as well as the depths of the tracks. Subsequently, based on TRACK_TEST, our group further developed the computer program TRACK_VISION [10] in 2008 to theoretically model the optical appearances of such tracks. These two computer programs were based on our proposed track development model, with some modifications [11]. The model involved two parameters—namely, the bulk etch rate *V_b_* and the track etch rate *V_t_*. For a homogeneous and isotropic detector material, the bulk etch rate *V_b_* is a constant for a specified etching condition (i.e., for the chosen etching chemical, chemical concentration, etching temperature, etc.). In contrast, *V_t_* generally varies along the trajectory of the incident ion that created the latent track and will vary according to the species and energy of the ion. The ratio *V_t_*/*V_b_* is referred to as the *V* function.

After the publication of the TRACK_TEST and TRACK_VISION programs [9,10], the idea was extended to studies on etched tracks developed in PADC films irradiated by protons [12]. The program TRACK_P calculated the track parameters and could also graphically provide two-dimensional and three-dimensional illustrations of proton tracks developed in the PADC films.

## 2. TRACK_TEST, TRACK_VISION and TRACK_P

TRACK_TEST enabled the calculations of track parameters in CR-39 and LR 115 SSNTDs, i.e., the lengths of the major and minor axes, as well as the depths of the alpha-particle tracks. Three and two built-in *V* functions were provided for the CR-39 SSNTD [13,14,15] and the LR 115 SSNTD [16,17], respectively. Based on the TRACK_TEST program, another program entitled TRACK_VISION was developed for visualizing the optical appearances of tracks developed in the chemically etched SSNTDs. Based on the geometrical optics, TRACK_VISION simulated light propagation through the tracks and computed the brightness of grid elements in the track wall [5]. A total of four built-in *V* functions were provided for the PADC detectors [13,14,15,18].

Since their publication, the programs TRACK_TEST and TRACK_VISION have been made available on the websites http://www.cityu.edu.hk/nru/Track_Test.htm and http://www.cityu.edu.hk/nru/Track_Vision.htm (accessed on 13 February 2021), respectively. As of 30 January 2021, the programs TRACK_TEST and TRACK_VISION have been cited 119 and 45 times, according to Google Scholar. Although there have been advancements in the field, such as the publication of new *V* functions, as well as continuous requests from various research groups for the creation of modifications and new options, e.g., for modeling for the Makrofol detector, the two programs TRACK_TEST and TRACK_VISION have not yet been updated or modified since their first publications. This current work is devoted to such updates and modifications.

## 3. Modifications of TRACK_TEST

### 3.1. Programming and Simulation

Detailed information on programming and simulation can be found in Reference [9], but for completeness, the basic points are briefly reviewed here. TRACK_TEST first determines the particle range in the detector and the depth *D* along the particle trajectory to which the etchant penetrates. Coordinates of points on the two-dimensional track wall for normal incidence are then computed, which are utilized to generate the track profile for non-normal incidence through the translation and rotation of the coordinate system [11]. A three-dimensional track is subsequently rendered through the rotation of the points on the two-dimensional track wall around the particle trajectory, from which the track opening contour, as well as the major and minor axes, are determined.

### 3.2. Adding Two V Functions

In this revised version of TRACK_TEST, two new *V* functions (expressed through the residual range *R*′, which is defined as the distance from a specific point on the particle track to its end point) were added and enabled. The first added *V* function was adopted from the work of Al-Jubbori [19] in the form

*V*(*R*′) = 1 + exp [–*a*_1_*R*′ + *a*_2_ − *a*_3_/*R*′ *+ a*_4_/*R*′*^a5^*], (1)

with the constants *a*_1_ = 0.098, *a*_2_ = 1.86, *a*_3_ = 37.78, *a*_4_ = 36.98 and *a*_5_ = 0.98 obtained by fitting the data provided by measuring the average lengths of the tracks (observed using an Optika B-193 microscope, Optika, Ponteranica, Italy) for different alpha-particle energies and etching times. In the revised TRACK_TEST, this function can be invoked by typing No. 4 when the program asks for the number of the *V* function.

The second added *V* function was adopted from the work of Hermsdorf [20] in the form

(2) $$V(R^{\prime })=1+\frac{a_{1}}{{\left(R^{\prime }+a_{2}\right)}^{b_{1}}}(1-e^{R^{\prime }/a_{4}})Ln(R^{\prime }+a_{3})+R^{\prime }/a_{5},$$

with constants *a*_1_ = 390, *a*_2_ = 2, *a*_3_ = 1, *a*_4_ = 5, *a*_5_ = 80 and *b*_1_ = 2.35. In the revised TRACK_TEST, this function can be invoked by typing No. 5 when the program asks for the number of the *V* function.

To summarize, together with the three built-in *V* functions originally provided in the previous version of TRACK_TEST for the CR-39 SSNTD, the five built-in *V* functions provided in the revised TRACK_TEST for the CR-39 SSNTD are:

No. 1 from Reference [13],

No. 2 from Reference [14],

No. 3 from Reference [15],

No. 4 from Reference [19] and

No. 5 from Reference [20].

**Figure 1:**
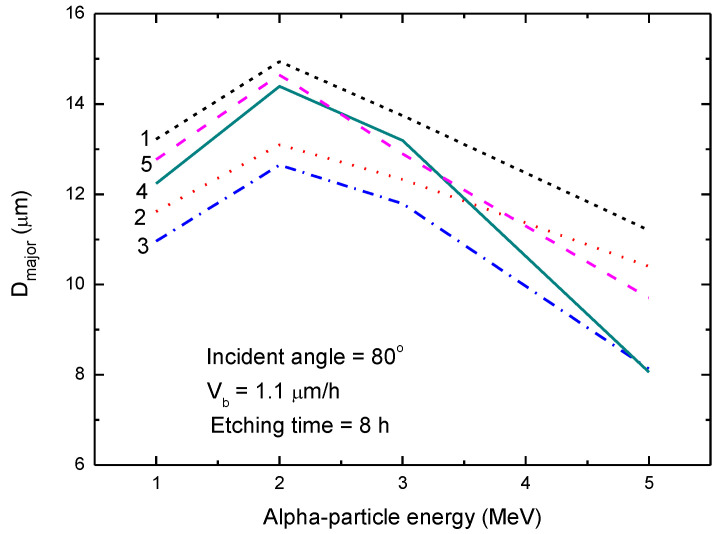
Relationship between the major-axis length (*D_major_*) of the track opening in the CR-39 detector and the alpha-particle energy for the incident angle of 80° for the five *V* functions implemented in the revised TRACK_TEST program. The experimental conditions are given in Figure. 1: *V* function No. 1 [13], 2: *V* function No. 2 [14], 3: *V* function No. 3 [15], 4: *V* function No. 4 [19] and 5: *V* function No. 5 [20]. Functions 4 and 5 were newly added in the revised TRACK_TEST program.

Figure 1 shows the relationship between the major-axis length (*D_major_*) of the track opening in the CR-39 detector and the alpha-particle energy for the incident angle of 80°. The results obtained using the five *V* functions are compared. Different results on *D_major_* were noticed. However, the discrepancies were not very significant. For example, for the incident alpha-particle energy of 1 MeV, all the results were in the range 12.2 ± 1.2 μm, which corresponded to discrepancies of only about 10%. For larger incident alpha-particle energies, the results were more scattered, in the range of 9.5 ± 1.3 μm, which, however, still corresponded to discrepancies of less than 14%.

### 3.3. Adding Makrofol Option

Another major revision of TRACK_TEST was to enable calculations for the Makrofol detector. In comparison to the CR-39 and LR115 detectors, there is, in general, a lack of data for the Makrofol detector. Even measurements on *V_b_* have not been common. One *V* function established for the Makrofol detector was given by Stajic et al. [21].

However, an unexpected behavior of this function was observed for low values of the residual range (i.e., the function was not equal to 1 when *R*′ → 0), so a slight modification was necessary to facilitate calculations by the computer programs. Furthermore, this *V* function led to numerical problems and incorrect plotting of the track profiles for over-etched tracks. These difficulties prompted us to find a better *V* function for Makrofol detectors without such drawbacks. The data obtained using the method described by Stajic et al. [21] by fitting to the new function:

(3) $$V\left(R^{\prime }\right)=1+\overset{5}{\underset{i=1}{\sum }}a_{i}R^{\prime b_{i}}e^{-c_{i}R^{\prime }}$$

gave fitting parameters *a*_i_, *b*_i_ and *c*_i_, given in Table 1. The coefficient of determination of the fit was *R*^2^ = 0.9998. In more detail, the data were calculated using the expressions involving the restricted energy loss [22,23], output data from SRIM-2013 [24] and the parameters provided by Somogyi et al. [23] for polycarbonate detectors. This new function was finally implemented in the modified programs in the present work.

**Figure 2:**
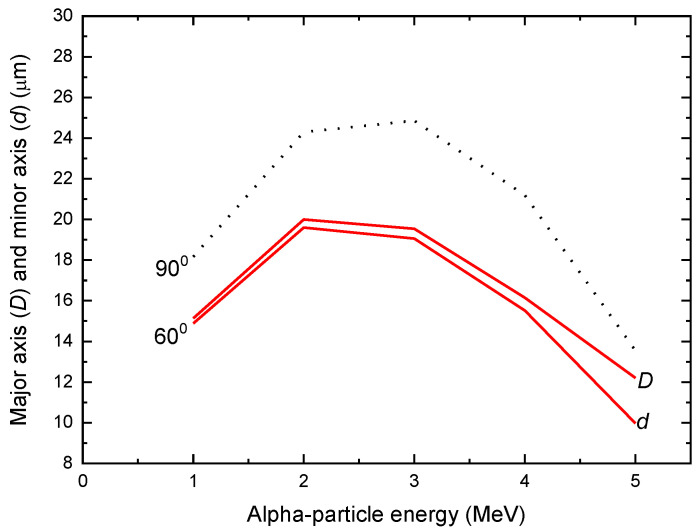
Relationships between the lengths of the major axis *D* and minor axis *d* of the elliptical track openings in the Makrofol detector and the alpha-particle energy for both incident angles of 90° and 60° for the PEW (potassium, ethanol and water) etchant (15-g KOH + 45-g H_2_O + 40-g ethyl alcohol), *V_b_* = 15 μm/h and 2 h of etching.

The bulk etch rate *V_b_* for the Makrofol detector was also determined by Stajic et al. [21]. For the PEW (potassium, ethanol and water) etchant (15-g KOH + 45-g H_2_O + 40-g ethyl alcohol), *V_b_* was found as 15 ± 1 μm/h. The relationships between the lengths of the major and minor axes of the track opening in the Makrofol detector and the alpha-particle energy are shown in Figure 2 for a removed layer of 30 μm and both incident angles of 60° and 90°.

## 4. Modifications of TRACK_VISION

### 4.1. Programming and Simulation

Detailed information on the programming and simulation can be found in Reference [10], but for completeness, the basic points are briefly reviewed here. TRACK_VISION simulated the appearance of tracks under the transparent mode of the optical microscope, where light rays came up from below the detector to strike the detector surface perpendicularly. The points on the three-dimensional track wall generated by TRACK_TEST were linked to form “four-angle polygons”, to which ray-tracing procedures were applied [25,26]. Any light ray exiting from the detector could then be characterized by its direction and relative intensity. The relative intensities for all elements on the three-dimensional track wall were then determined.

### 4.2. Adding Two V Functions and Makrofol Option

**Figure 3:**
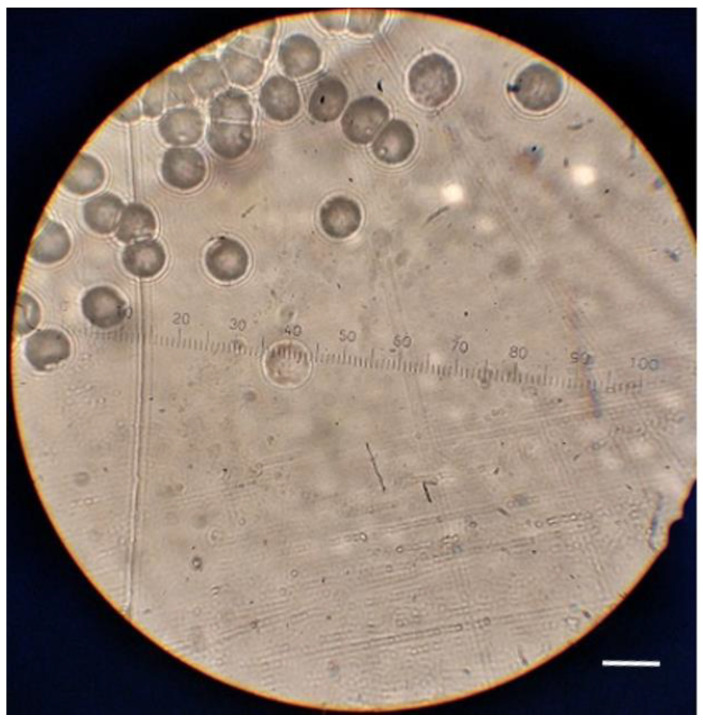
Alpha-particle tracks developed on an irradiated and etched Makrofol detector captured using an optical microscope. The detector was irradiated with alpha particles with an energy of 3.6 MeV under normal incidence and was then etched for 2 h. The removed detector thickness was determined as 30 ± 2 μm. White scale bar: 26 µm.

The TRACK_VISION program was also modified to accommodate the two new *V* functions for PADC films and to provide the new option for calculations for the Makrofol detector. Both new *V* functions for PADC detectors described in Equations (1) and (2) were programmed. A simulation of the optical appearance of tracks developed in the Makrofol detector was enabled. A sample image of the alpha-particle tracks developed on an irradiated and etched Makrofol detector captured using an optical microscope is shown in Figure 3. The detector was irradiated with alpha particles with an energy of 3.6 MeV under normal incidence (using an electroplated ^241^Am source and a collimator with an appropriate thickness). The incident energy (3.6 MeV) was estimated based on the distance in the air between the source and the detector (i.e., the collimator thickness) and a function obtained by fitting SRIM-2013 output data for helium in the air. The detectors were etched for 2 h, and the removed detector thickness was determined as 30 ± 2 μm.

**Figure 4:**
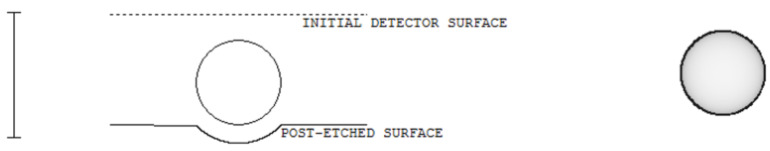
Simulated results obtained by the revised TRACK_VISION program corresponding to those alpha-particle tracks developed on an irradiated and etched Makrofol detector shown in Figure 3. Left panel: track profile and track opening (the latter being a circle due to normal incidence), together with the position of the detector surface before and after etching. Right panel: optical track appearance, which consists of a wide, dark ring and a bright area at the track center. Vertical left bar represents 34 μm.

Corresponding to those alpha-particle tracks developed on an irradiated and etched Makrofol detector shown in Figure 3, the simulated results obtained by the revised TRACK_VISION program are shown in Figure 4. The left panel of Figure 4 shows the track profile and track opening (the latter being a circle for normal incidence) simulated using the revised TRACK_VISION, together with the position of the detector surface before and after etching. On the other hand, the right panel of Figure 4 shows the corresponding optical track appearance. The diameter of the experimentally obtained alpha-particle tracks presented in Figure 3 was estimated to be 24.7 ± 1.4 µm with the help of the scale in Figure 3. The diameter given by the revised TRACK_VISION program was 23.2 μm, and the two values were deemed to have good agreement.

As regards the optical appearance of the tracks, there were similarities and differences between the simulated and experimental results. Notably, a bright area was present in the central parts of both simulated and experimental tracks, which was explained by the light passing through the tracks without a significant loss of intensity. Interestingly, however, the appearances of the tracks close to their perimeters were different for the simulated and experimental tracks. The simulated track shown in the right panel in Figure 4 showed an outer dark ring caused by a total reflection of the light coming from the bottom of the detector. In the experimental results shown in Figure 3, dark rings were also observed close to the perimeters of the tracks but were enclosed by bright interference fringes. It is remarked here that the interference of light was not taken into account in the present work, where only the geometrical optics were considered. The incorporation of wave optics will be carried out in our future works.

## 5. Notes on Installing and Running TRACK_TEST 2.0 and TRACK_VISION 2.0

The revised TRACK_TEST and TRACK_VISION programs are referred to as TRACK_TEST 2.0 and TRACK_VISION 2.0. These programs are freeware downloadable from the websites http://www.cityu.edu.hk/nru/Track_Test.htm and http://www.cityu.edu.hk/nru/Track_Vision.htm (accessed on 13 February 2021), respectively, and are compressed in ZIP format. After downloading, the files for a chosen program (TRACK_TEST 2.0 or TRACK_VISION 2.0) should be extracted and stored in a directory created by the user. Note that different directories should be created for files from the two different programs. Further compilation is not needed. When a program is started, it will read input data from the file INPUT.DAT, which can be edited using any text editor. The programs enable calculations for three kinds of the most frequently used detectors—namely, the CR-39 detector and the LR 115 detector, as well as the Makrofol detector. The user can choose the detector by typing “C” for CR-39, “L” for LR 115 and “M” for Makrofol in INPUT.DAT. More detailed instructions on running the programs can be found on their respective webpages.

Upon successful running of the programs, the lengths of the major and minor axes of the track opening, as well as the track depth, are given. The following will also be shown on the computer screen: initial detector surface, detector surface after etching, track profile, contour of track opening and trajectory of the particle, as well as the scale for determining the real dimensions from the images. The aspect ratio of the screen should be set to 4:3 in order to show undistorted images. For TRACK_VISION 2.0, the simulated appearance of a track under the transparent mode of the optical microscope will also be displayed.

## 6. Conclusions

The current work provided updates and modifications to two previously developed computer programs: TRACK_TEST and TRACK_VISION.

The computer program TRACK_TEST was first developed in 2006 [9] to model alpha-particle track profiles that enable the determination of parameters including the lengths of major and minor axes, as well as the depths of the tracks. In the revised version of TRACK_TEST, two *V* functions were added, which were taken from the research works of Al-Jubbori [19] and Hermsdorf [20]. Moreover, the Makrofol option was added by adopting the *V* function from Stajic et al. [21], with some modifications. To differentiate between the original and revised versions of TRACK_TEST, the current revised version is entitled TRACK_TEST 2.0, and both the original version TRACK_TEST and the revised version TRACK_TEST 2.0 are available on the website http://www.cityu.edu.hk/nru/Track_Test.htm (accessed on 13 February 2021).

The computer program TRACK_VISION was developed in 2008 [10] to model the optical appearances of such tracks. In the revised version of TRACK_VISION, the two *V* functions for the CR-39 detector established by Al-Jubbori [19] and Hermsdorf [20] were also added, and the Makrofol option was also enabled. To differentiate between the original and revised versions of TRACK_VISION, the current revised version is entitled TRACK_VISION 2.0, and both the original version TRACK_VISION and the revised version TRACK_VISION 2.0 are available on the website http://www.cityu.edu.hk/nru/Track_Vision.htm (accessed on 13 February 2021).

## Figures and Tables

**Table 1:** Parameters of the *V* function *V*(*R*′) for the Makrofol detector described in Equation (3).

	1	2	3	4	5
*a*	0.1744	−0.0672	0.3396	0.0921	1761.3219
*b*	1.6652	1.2607	4.2000	1.1676	50.0726
*c*	0.3549	0.0460	1.4294	0.0444	23.0569

## Data Availability

The computer program TRACK_TEST 2.0 is available on the website http://www.cityu.edu.hk/nru/Track_Test.htm (accessed on 13 February 2021). The computer program TRACK_VISION 2.0 is available on the website http://www.cityu.edu.hk/nru/Track_Vision.htm (accessed on 13 February 2021). All experimental data were reported in this paper.

## References

[1] Abu-Jarad F.A. (1988). Application of nuclear track detectors for radon related measurements. Int. J. Radiat. Appl. Instrum..

[2] Nikolaev V.A., Ilic R. (1999). Etched track radiometers in radon measurements: A review. Radiat. Meas..

[3] Steck D.J., Field R.W. (1999). The use of track registration detectors to reconstruct contemporary and historical airborne radon (^222^RN) and radon progeny concentrations for radon-lung cancer epidemiologic study. Radiat. Meas..

[4] Falk R., Almrén K., Östergren I. (2001). Experience from retrospective radon exposure estimations for individuals in a radon epidemiological study using solid-state nuclear track detectors. Sci. Total Environ..

[5] Bochicchio F. (2005). Radon epidemiology and nuclear track detectors: Methods, results and perspectives. Radiat. Meas..

[6] Yu K.N., Nikezic D., Ng F.M.F., Leung J.K.C. (2005). Long-term measurements of radon progeny concentrations with solid state nuclear track detectors. Radiat. Meas..

[7] Yu K.N., Nikezic D. (2011). Long-term determination of airborne radon progeny concentrations using LR 115 solid-state nuclear track detectors. Radiat. Meas..

[8] Nikezic D., Yu K.N. (2004). Formation and growth of tracks in nuclear track materials. Mater. Sci. Eng. R..

[9] Nikezic D., Yu K.N. (2006). Computer program TRACK_TEST for calculating parameters and plotting profiles for etch pits in nuclear track materials. Comput. Phys. Commun..

[10] Nikezic D., Yu K.N. (2008). Computer program TRACK_VISION for simulating optical appearance of etched tracks in CR-39 nuclear track detectors. Comput. Phys. Commun..

[11] Nikezic D., Yu K.N. (2003). Three-dimensional analytical determination of the track parameters. Over-etched tracks. Radiat. Meas..

[12] Nikezic D., Ivanovic M., Yu K.N. (2016). A computer program TRACK_P for studying proton tracks in PADC detectors. SoftwareX.

[13] Durrani S.A., Bull R.K. (1987). Solid State Nuclear Track Detection. Principles, Methods and Applications.

[14] Brun C., Fromm M., Jouffroy M., Meyer P., Groetz J.E., Abel F., Chambaudet A., Dorschel B., Hermsdorf D., Bretschneider R. (1999). Intercomparative study of the detection characteristics of the CR-39 SSNTD for light ions: Present status of the Besancon—Dresden approaches. Radiat. Meas..

[15] Yu K.N., Ng F.M.F., Nikezic D. (2005). Measuring depths of sub-micron tracks in a CR-39 detector from replicas using atomic force microscopy. Radiat. Meas..

[16] Durrani S.A., Green P.F. (1984). The effect of etching conditions on the response of LR 115. Nucl. Tracks.

[17] Yip C.W.Y., Nikezic D., Ho J.P.Y., Yu K.N. (2006). Chemical etching characteristics for cellulose nitrate. Mater. Chem. Phys..

[18] Yu K.N., Ho J.P.Y., Nikezic D., Yip C.W.Y., Mendez-Vilas A. (2005). Determination of the V function for CR-39 by atomic force microscope. Recent Advances in Multidisciplinary Applied Physics.

[19] Al-Jubbori M.A. (2020). V-function to investigate tracks of the alpha particle irradiated CR-39 detector. Radiat. Meas..

[20] Hermsdorf D. (2009). Evaluation of the sensitivity function V for registration of α-particles in PADC CR-39 solid state nuclear track detector material. Radiat. Meas..

[21] Stajic J.M., Milenkovic B., Nikezic D. (2018). Study of CR-39 and Makrofol efficiency for radon measurements. Radiat. Meas..

[22] Benton E.V., Nix W.D. (1969). The restricted energy loss criterion for registration of charged particles in plastics. Nucl. Instrum. Methods.

[23] Somogyi G., Grabisch K., Scherzer R., Enge W. (1976). Revision of the concept of registration threshold in plastic track detectors. Nucl. Instrum. Methods.

[24] Ziegler J.F., Ziegler M.D., Biersack J.P. (2010). SRIM: The stopping and range of ions in matter. Nucl. Instrum. Methods Phys. Res. B.

[25] Nikezic D., Ng F.M.F., Yip C.W.Y., Yu K.N. (2005). Application of ray tracing method in studying alpha tracks in SSNTDs. Radiat. Meas..

[26] Yu K.N., Lee H.H.W., Wong A.W.T., Law Y.L., Cheung S.F.L., Nikezic D., Ng F.M.F. (2007). Optical appearance of alpha-particle tracks in CR-39 SSNTD. Nucl. Instrum. Methods Phys. Res. B..

